# Retroperitoneal Laparoscopic Ureterolithotomy for Proximal Ureteral Calculi in Selected Patients

**DOI:** 10.1155/2014/687876

**Published:** 2014-12-08

**Authors:** Qingfeng Hu, Weihong Ding, Yuancheng Gou, Yatfaat Ho, Ke Xu, Bin Gu, Chuanyu Sun, Guowei Xia, Qiang Ding

**Affiliations:** ^1^Department of Urology, Huashan Hospital, Fudan University, No. 12 Central Urumqi Road, Shanghai 200040, China; ^2^Fudan Institute of Urology, Huashan Hospital, Fudan University, Shanghai 200040, China; ^3^Department of Surgery, Shanghai Medical College, Fudan University, Shanghai 200040, China

## Abstract

*Objectives*. To summarize our experience of retroperitoneal laparoscopic ureterolithotomy for ureteral calculi and evaluate the safety and efficiency of this procedure. *Methods*. We conducted a retrospective analysis of 197 patients with proximal ureteral calculi who accepted retroperitoneal laparoscopic ureterolithotomy from June 2005 to June 2014. *Results*. All procedures were performed successfully and the mean operating time and estimated blood loss were 87 min and 64 mL. The clearance rate was 98.5% and the rates of urine leak and ureteral stricture were 2.5% and 1.0%. *Conclusions*. Retroperitoneal laparoscopic ureterolithotomy is a safe and effective procedure for patients with complex stones or anatomic abnormalities, and, with experience of high volume series, it is also a reasonable choice as the primary treatment for such selected patients.

## 1. Introduction

Urolithiasis is an extremely common disease, and the management of ureteral calculi has been changing since the appearance of shockwave lithotripsy (SWL), ureterorenoscopy (URS), and percutaneous nephrolithotomy (PNL). The aim of treatment is to achieve a stone-free status and protect the ipsilateral renal function as soon, as safely, and as minimally invasively as possible. However, the best treatment is still controversial, extremely for patients with complex ureteral stones or anatomic abnormalities. SWL should not be recommended as the first-line treatment option for the management of large ureteral stones with severe hydronephrosis [1]. And, with clearance rates of 60–90%, URS and PNL could not obtain satisfactory results yet [2–4].

Ureterolithotomy is often accepted as an ultimate means for the most difficult stone and always occupies its place although SWL, URS, and PNL have brought about the revolutionary change to the management of proximal ureteral calculi [5]. Laparoscopy has provided a novel option for the replacement of conventional open surgery, and the advantages on analgesia, recuperation, hospital stay, and cosmetics over open surgery are obvious [6]. Although laparoscopic ureterolithotomy (LU) is not the first choice in most cases for its invasiveness, LU has the highest stone-free rate (SFR) compared to SWL and URS, and LU also has its unique advantages on patients with large impacted stone, severe hydronephrosis, or anatomic anomalies [7]. From our preliminary experience, we have recognized its potential and advantages in selected patients. Different from traditional consensus, LU could be accepted as a preferred choice and get effective clearance with fast recovery.

## 2. Materials and Methods

### 2.1. Enrolled Patients

**Figure 1:**
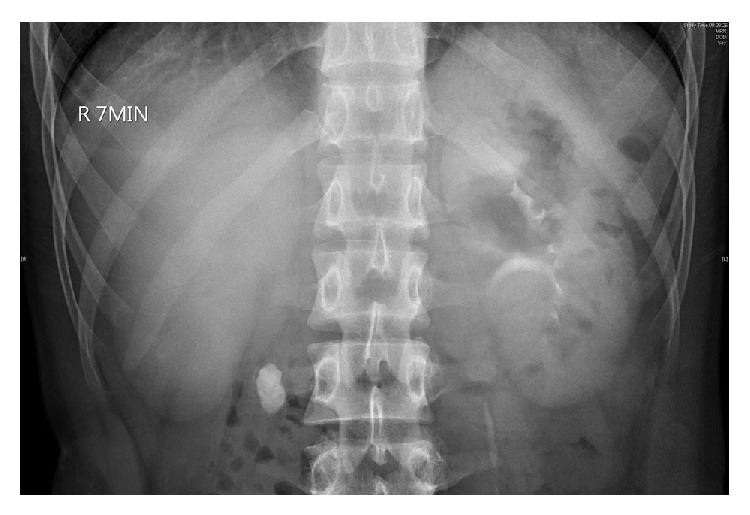


A series of 197 patients with proximal ureteral calculi who accepted retroperitoneal LU in our center, Department of Urology, Huashan Hospital of Fudan University, from June 2005 to June 2014 were enrolled. The following information was collected retrospectively: gender, age, side and size of ureteral stones, operating time, blood loss, postoperative hospital stay, and complications. The clearance rate was also an important indicator to evaluate the result of LU, which was defined as the status of no residual stone in IVU and CT. All patients got the definite diagnosis before operation, from the results of ultrasonography, intravenous urography (IVU), and computed tomography (CT) (Figure 1). Not all patients would experience colic. Patients with one of the following indications would be considered to be suitable for LU: (a) proximal ureteral calculi larger than 1.5 cm; (b) distal ureteral stricture with ureteral polypus or external compression. LU could also be considered for patients who suffered ureteral stones no larger than 1.5 cm but severe hydronephrosis without self-discharge for long course in follow-up, especially for those with abnormal anatomy or prior failure of treatment with SWL or URS. Calculi locating in proximal ureters which were from ureteropelvic junction to the upper edge of the pelvis would be considered to be treated with LU. The coinstantaneous renal stones were not treated in the same operation. When a patient suffered multiple ureteral stones in the same location, LU could be used to clear all visible stones. However, when a patient suffered multiple ureteral stones in different locations, LU would be used to clear the largest one, and the others could be cleared by SWL or URS. All patients were informed to follow up 1 to 3 months after operation. Ultrasonography and CT could be used to evaluate the hydronephrosis compared to that before operation; X-ray and IVU could be used to evaluate the residual status of stone and potential ureteral stricture after operation.

### 2.2. Operative Technique

**Figure 2:**
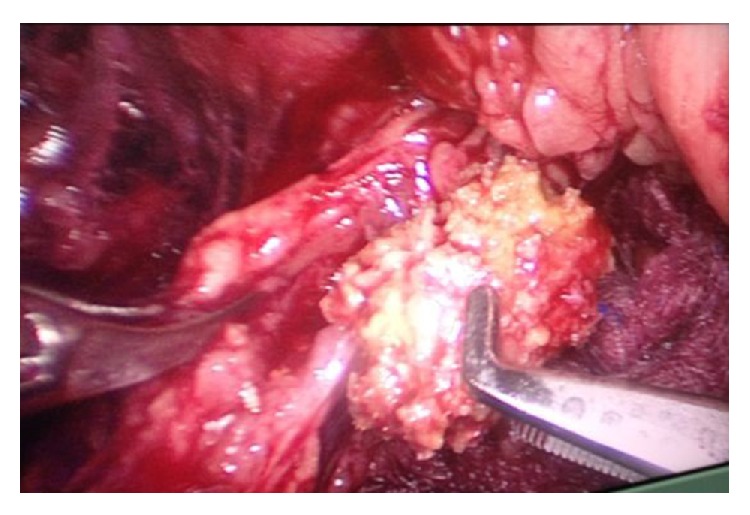


All the operations were performed by one experienced surgeon (Guowei Xia) who did well in urologic laparoscopic surgery. LU was conducted by the conventional three ports procedure under a lateral decubitus position with hyperextension, which was reported in our previous similar study [8]. To establish working space, the fascia lumbodorsalis was divided by a hemostatic forceps through a 2 cm incision over the iliac crest, and the retroperitoneal fat and the retroperitoneal space were separated by the digital dissection and balloon dilatation. Two ports were guided by index finger and placed at the subcostal anterior and posterior axillary line, and the port of camera was at the former incision over the iliac crest. The ureteral stone could be identified along the bulge of ureter in the area anterior to the psoas major muscle after opening Gerota's fascia and renal capsule. The ureteral stone was extracted from a longitudinal incision, and a 6F D-J stent (Cook Medical) was inserted routinely (Figure 2). The method used to insert D-J stent was similar to that mentioned by Fan and colleagues [9]. Then the ureteral incision could be closed by interrupted sutures. A retroperitoneal drain was inserted and removed once it was below 20 mL, and the D-J stent would be extracted 3-4 weeks later by cystoscopy.

## 3. Results

The characteristics of patients and perioperative data were listed in Table 1. The mean size of ureteral calculi was 22 mm, and all procedures were performed in laparoscopy with no conversion to open surgery. The average of operating time and estimated blood loss was 87 min and 64 mL and that of hospital stay and retroperitoneal drain was 3.6 days and 2.7 days (patients with urine leak excluded).

The clearance rates at discharge and four weeks later were 97.5% and 98.5%. Three patients were found to have ureteral stones that migrated back to pelvis during operation and they accepted PNL later. The rates of urine leak and postoperative ureteral stricture were 2.5% (5/197) and 1.0% (2/197). To those with urine leak, the indwelling period of retroperitoneal drain and D-J stent should extend, respectively, until recovery after about 6 weeks, and to those with postoperative ureteral stricture, they accepted ureteroscopy and dilatation three months after LU.

## 4. Discussion

The treatment of ureteral calculi has evolved in recent decades and the ultimate objects are clearance and minimal invasion. Current options including SWL and URS have the particular rates of clearance, complications, and limitations, respectively. However, large and impacted proximal stone is still a tricky problem whose treatment remains controversy. There is no doubt that SWL is the least invasive procedure, and the success rate is not that satisfactory. It is reported that the clearance rate after a single session of SWL would decrease from 83.6% to 42.1% when the stone is larger than 10 mm [10]. Although the improvement of equipment and technology enhances the ability of URS in the treatment of ureteral calculi, URS is still limited by its clearance rate and complications. An overall complication rate after URS is about 25% [11]; proximal location and stone impaction are common factors predicting unfavorable results [12].

Ureterolithotomy always has its place in the treatment of ureteral calculi, and laparoscopic surgery is increasingly replacing open surgery today. Although with higher morbidity than SWL and URS, LU could still be considered as a minimally invasive procedure. LU has its particular advantage in those with most complex stones, previous failed treatment of SWL or URS, and anatomical abnormalities. It is the most important that LU has the highest SFR compared to SWL and URS for proximal ureteral calculi [7]. According to our data and other published high volume studies, the advantage is obvious (Table 2) [2, 4, 13–23]. The procedure of LU is more complex than SWL and URS, and the operating duration is longer than SWL and URS [3]. However, LU seems to be not difficult and is associated with a short learning curve [9]. LU could achieve a high level of efficacy with low rates of complication. It is the main aspect of morbidity for LU to break the intraperitoneal or retroperitoneal structures and integrity of collecting system. It would be still considered less serious than the trauma from major complications in URS, such as ureteral perforation and ureteral avulsion, nevertheless. To try SWL or URS in every patient is meaningless and hazardous; it is more reasonable for patients to choose appropriate treatment depending on the situation.

The common and major complications of LU include stone migration, urine leak, and ureteral stricture. However, with low rates of complications, LU is an effective and safe procedure (Table 3). To confirm the location of stone, an X-ray of kidney, ureter, and bladder (KUB) is needed routinely, and dissection of the ureter from the bulge to the stone site should be performed carefully to avoid stone migration. The appearance of migration often means failure of LU; open conversion or postponed PNL would increase the morbidity. A more common complication is urine leak, which is in the form of prolonged drainage and elevated creatine in it. We have experienced 5 cases, particularly in those with long course and inflamed edematous ureter. Placing a D-J stent plays an important role in prevention while it does not increase the duration of operation, and it should be accepted as a routine procedure [24]. Another complication is ureteral stricture, relatively. Stitching too tight or too loose is a common problem encountered by beginners; looseness is associated with urine leak while tightness is associated with ureteral stricture. Our experience implies that loose stitching should be more appropriate. Under the existence of D-J stent, most cases of urine leak would close up spontaneously in a prolonged period.

It is an interesting issue whether transperitoneal LU or retroperitoneal LU is more dominant. Generally, both transperitoneal LU and retroperitoneal LU are effective, safe, and feasible, and the choice depends on personal preference and experience of surgeons [25]. Here, we prefer retroperitoneal LU, based on former experience in open and laparoscopic surgery for renal and ureteral diseases. Even more important, we believe that retroperitoneal LU would be associated with less morbidity and faster recovery, which would influence less intraperitoneal tissue and organs. Besides, transperitoneal approach would bring about the adhesion and modification of normal anatomy and create difficulties for possible intraperitoneal operations in the future. As a minimally invasive procedure, laparoscopic surgery is superior in the less influence and destruction of normal anatomic structure compared to open surgery, and so it is with retroperitoneal procedure to transperitoneal procedure.

Our study also has its limitations as a retrospective series in a single center. With lack of comparison, these results are not representative. SWL and URS are more likely to be accepted and are recommended as main methods for the treatment of ureteral calculi. Although LU could also provide satisfactory results, increased complexity and trauma would limit its application. In fact, considering the techniques and experience, together with the patients' conditions, the appropriate method is the best for patients. Additionally, we have just focused on the short-term prognosis of LU and have been short of long-term results. LU causes destruction for the normal structure of ureter after all. The long-term outcomes of LU remain unclear, which need further study to give robust conclusions.

## 5. Conclusions

As a safe and effective procedure for ureteral calculi, retroperitoneal LU is suitable for patients with complex stones or anatomic abnormalities and provides the highest SFR in the treatment of ureteral calculi. Indeed, with experience of high volume series, it is also a reasonable choice as the primary treatment for such selected patients.

## Figures and Tables

**Table 1:** The characteristics of patients and perioperative data.

Sex (male/female)	116/81
Mean age (y) (range)	41 (20–73)
Side of disorder (left/right)	89/108
Mean size of ureteral calculi (mm) (range)	22 (14–35)
Indications	
Large stones	139 (70.5)
Abnormal anatomy or ureteral stricture	47 (23.9)
Severe hydronephrosis or prior failure of treatment with SWL or URS	11 (5.6)
Mean operating time (min) (range)	87 (65–165)
Mean estimated blood loss (mL) (range)	64 (20–150)
Postoperative hospital stay (d) (range)	3.6 (2–7)
Clearance in follow-up of four weeks (%)	194 (98.5)
Complications	
Migration back to pelvis of calculi (%)	3 (1.5)
Urine leak (%)	5 (2.5)
Ureteral stricture (%)	2 (1.0)

**Table 2:** Perioperative data of laparoscopic ureterolithotomy for ureteral calculi.

Authors	Year	Cases	Mean stone size (mm)	Mean operating time (min)	Postoperative hospital stay (d)	Stone-free rate (%)
Flasko et al. [13]	2005	73	25	45	3.3	100
Kijvikai and Patcharatrakul [14]	2006	30	19	121.4	3.9	100
El-Feel et al. [15]	2007	25	19	145	4.1	100
El-Moula et al. [16]	2008	74	18	58.7	6.4	100
Huri et al. [17]	2010	41	22	124	4.8	100
Wang et al. [18]	2010	36	17.3	131.5	5.8	100
Farooq Qadri et al. [19]	2011	126	13.6	88	2.8	97.6
Leonardo et al. [20]	2011	33	34	85	3.4	100
Ko et al. [21]	2011	32	18.1	117.8	5.9	93.8
Basiri et al. [2]	2008	50	22.4	127.8	5.8	90
Karami et al. [22]	2013	40	13.5	82	3.5	100
Nasseh et al. [23]	2013	33	—	85.5	4.1	93.9
Zhu et al. [4]	2014	21	15	93.7	6.1	90.5
Current experience	2014	197	22	87	3.6	98.5

**Table 3:** Complications of laparoscopic ureterolithotomy for ureteral calculi.

Authors	Year	Cases	Stone migration (*n*, %)	Urine leak (*n*, %)	Ureteral stricture (*n*, %)
Flasko et al. [13]	2005	73	—	6, 8.2	0, 0
El-Moula et al. [16]	2008	74	1, 1.4	1, 1.4	1, 1.4
Huri et al. [17]	2010	41	1, 2.4	5, 12.2	1, 2.4
Farooq Qadri et al. [19]	2011	126	—	3, 2.4	—
Leonardo et al. [20]	2011	33	0, 0	1, 3.0	0, 0
Ko et al. [21]	2011	32	2, 6.3	—	1, 3.1
Karami et al. [22]	2013	40	—	3, 7.5	—
Nasseh et al. [23]	2013	33	1, 3.0	0, 0	0, 0
Current experience	2014	197	3, 1.5	5, 2.5	2, 1.0
